# Harnessing nanoprodrugs to enhance cancer immunotherapy: overcoming barriers to precision treatment

**DOI:** 10.1016/j.mtbio.2025.101933

**Published:** 2025-05-31

**Authors:** Yunfan Lin, Pei Lin, Xu Chen, Xinyuan Zhao, Li Cui

**Affiliations:** a Stomatological Hospital, School of Stomatology, Southern Medical University, Guangzhou, 510280, Guangdong, China; b School of Dentistry, University of California, Los Angeles, Los Angeles, 90095, CA, USA

**Keywords:** Nanoprodrug, Cancer immunotherapy, Tumor microenvironment

## Abstract

Nanoprodrugs, leveraging advanced nanoparticle-based delivery systems, represent a promising strategy to enhance the efficacy of immunotherapy in cancer treatment. These systems offer precise tumor targeting, controlled drug release, and the potential to modulate the immune microenvironment, addressing several limitations of conventional therapeutic approaches. This review systematically evaluates the role of nanoprodrugs in improving immunotherapy outcomes, focusing on their ability to overcome challenges such as poor bioavailability, systemic toxicity, and limited tumor specificity. We also discuss the key advantages of these systems, including their ability to co-deliver immune checkpoint inhibitors and other immunomodulatory agents, potentially enabling more synergistic and effective treatment strategies. Despite their promise, several challenges remain, including achieving precise control over drug release, integrating multiple stimulus-responsive mechanisms, addressing tumor heterogeneity, and overcoming barriers to clinical translation. The review concludes with a perspective on future directions, emphasizing the need for further optimization of nanomaterial design, improved delivery strategies, and solutions to the complexities of the tumor microenvironment to maximize the clinical impact of nanoprodrugs in cancer immunotherapy.

## Graphical abstract

This schematic illustrates the mechanisms by which various nanoprodrugs, including inorganic, organic, hybrid, biological, and carrier-free systems, enhance cancer immunotherapy through immune activation. Nanoprodrugs, by inducing immunogenic cell death (ICD), release tumor-associated antigens (TAAs) and damage-associated molecular patterns (DAMPs), which activate dendritic cells (DCs) and stimulate T cell-mediated immune responses. Additionally, nanoprodrugs can modulate immune checkpoints such as the PD-1/PD-L1 pathway, overcoming immune suppression and restoring T cell function to promote anti-tumor immunity. The activation of the cGAS-STING pathway by nanoprodrugs enhances the production of type I interferons, which further stimulate DC activation and the infiltration of CD8^+^ T cells into tumors. Nanoprodrugs also target immune-suppressive factors such as IDO and TGF-β, boosting T cell activity. Furthermore, nanoprodrugs can reprogram tumor-associated macrophages (TAMs) from an immunosuppressive M2 phenotype to a pro-inflammatory M1 phenotype, increasing the immune response. Together, these effects facilitate the transition from cold tumors, which are characterized by limited immune infiltration, to hot tumors, where effective immune activation and anti-tumor immunity occur.

**Figure:**
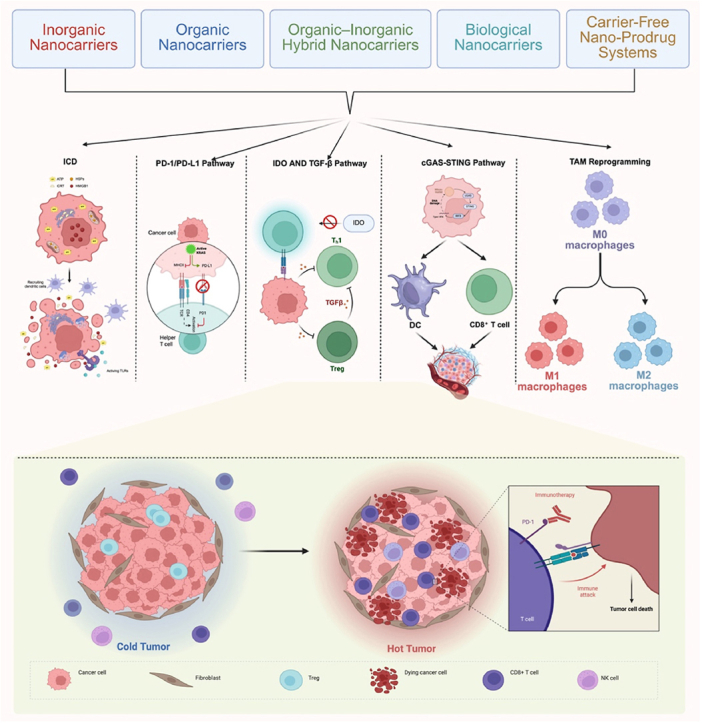

## 1 Introduction

Immunotherapy represents a transformative approach in cancer treatment, offering the potential to leverage the body's immune system to target and eradicate malignant cells. While it has demonstrated promising efficacy in certain cancers, significant barriers remain that limit its broader success. These include resistance to immune checkpoint inhibitors, the presence of immunosuppressive tumor microenvironments, and challenges in effectively delivering immune-modulatory agents to tumor sites. Overcoming these hurdles to improve the specificity, potency, and durability of immune responses is therefore essential to advancing the clinical utility of immunotherapy [[1], [2], [3]].

A promising strategy to address these challenges is the use of nanotechnology for the targeted delivery of prodrugs. Nanoparticle-based systems allow for the precise, controlled release of therapeutic agents, improving their bioavailability, minimizing off-target toxicity, and enhancing tumor-specific targeting. By encapsulating prodrugs within nanoparticles, these systems not only protect the drugs from premature degradation but also facilitate their sustained and localized activation within the tumor microenvironment. This approach offers significant potential to overcome key obstacles in immunotherapy, particularly by improving drug delivery and modulating immune responses in a more controlled and efficient manner [4]. For instance, a hybrid nanomedicine combining a porphyrin metal-organic framework (MOF) and gold nanoparticles (AuNPs) enhances tumor-targeted delivery and stability of the hypoxia-activated prodrug tirapazamine (TPZ). The nanomedicine releases TPZ in response to intracellular phosphate, exacerbating tumor hypoxia during PDT. This synergistic approach activates chemotherapy under hypoxic conditions, significantly improving antitumor efficacy and metastasis inhibition, offering a promising strategy for enhanced cancer treatment [5]. In addition, the hypoxia-directed nanosensitizer formulation encapsulates the hypoxia-activated prodrug iodoazomycin arabinofuranoside (IAZA) in a carbohydrate-based nanogel, enhancing selective drug delivery to hypoxic tumor cells. The thermoresponsive nanogel, with a galactose-based shell, ensures high IAZA loading and controlled release. NanoIAZA demonstrates superior hypoxia-selective cytotoxicity and radiosensitization in head and neck and prostate cancer cell lines, improving tumor regression and survival in xenograft models [6].

This review comprehensively examines the use of nanoprodrugs in enhancing immunotherapy, focusing on nanoparticle-based systems designed to improve drug delivery and overcome existing therapeutic limitations. We also discuss the advantages of nanoparticle systems for nanoprodrug delivery, including targeted drug release, enhanced bioavailability, and the ability to co-deliver immunomodulatory agents for synergistic effects. Finally, we address the challenges and future directions of nanoprodrugs in immunotherapy, emphasizing issues with scalability, immunogenicity, and tumor targeting, and suggest strategies to optimize these systems for clinical use.

## 2 Immunotherapy and tumor immune evasion

Immunotherapy harnesses the immune system to combat diseases, particularly cancer, by employing strategies such as immune checkpoint inhibitors (ICIs), cellular therapies, and vaccine therapies. ICIs act by inhibiting proteins that cancer cells exploit to suppress immune surveillance, thereby reactivating T cells to effectively recognize and eliminate tumors [7]. Cellular therapies, such as CAR T-cell therapy, involve the ex vivo genetic engineering of a patient's immune cells to enhance their ability to identify and eradicate cancer cells, specifically programming T cells to target cells presenting specific tumor antigens [8]. Vaccine therapies, meanwhile, aim to prime the immune system by introducing cancer-specific antigens that elicit a robust immune response, enabling the detection and destruction of malignant cells [9]. Each of these approaches capitalizes on advanced biotechnological methodologies to amplify the immune system's innate capacity to target and eliminate malignancies, offering significant promise for cancer treatment.

Despite the promise of these therapies in controlling tumor growth, cancer cells frequently develop sophisticated mechanisms to evade immune detection and destruction, posing significant challenges to therapeutic efficacy. By modifying their microenvironment, tumor cells create physical and chemical barriers that hinder effective immune surveillance and response. A common evasion strategy involves altering antigen presentation; tumor cells may downregulate major histocompatibility complex (MHC) molecules, essential for T-cell recognition, effectively rendering themselves "invisible" to immune surveillance. IRGQ regulates MHC class I quality control by directing misfolded MHC I molecules to lysosomal degradation via interactions with GABARAPL2 and LC3B. Loss of IRGQ leads to the accumulation of MHC I on the cell surface, enhancing immune recognition. In hepatocellular carcinoma, reduced IRGQ levels increase CD8^+^ T cell reactivity and improve survival, promoting antitumor immunity [10]. Concurrently, they often upregulate immune checkpoint proteins, such as PD-L1, which bind to PD-1 on T cells, delivering inhibitory signals that suppress T-cell function and proliferation. For instance, IFNα upregulates PD-L1 by inducing TRIM14, which recruits USP14 to prevent PD-L1 degradation via autophagy. This enhances PD-L1 surface expression, inhibiting CD8^+^ T cell antitumor activity [11].

The tumor microenvironment (TME) plays a pivotal role in facilitating immune evasion by tumor cells. It secretes a variety of cytokines and chemokines that recruit immunosuppressive cells, including regulatory T cells (Tregs) and suppressor cells (MDSCs). These cells establish an immunosuppressive milieu, undermining the efficacy of effector immune cells [[12], [13], [14]]. Depletion of Arg1^+^ TAMs reduces TH1-Treg cells in the TME by inhibiting PF4 secretion. PF4 promotes IFN-γ-induced TH1-Treg polarization, and its neutralization or genetic inactivation suppresses tumor growth [15]. Furthermore, tumor cells induce changes within the TME, impairing the function and survival of infiltrating immune cells while promoting tumor adaptation and survival under these adverse conditions. For instance, TGM2 promotes T cell suppression in PDAC by modulating microtubule dynamics and vesicle trafficking, facilitating the secretion of immunosuppressive cytokines. Targeting TGM2 or disrupting microtubules enhances T cell-mediated cytotoxicity, offering a potential strategy to overcome immune resistance in PDAC [16]. Resistance to therapies, including ICIs, may also arise as tumor cells acquire mutations in targeted pathways or activate alternative signaling mechanisms to circumvent the effects of treatment. KRAS-driven tumor growth is primarily mediated by the ERK MAPK pathway, which regulates key genes and cell cycle machinery, including the anaphase promoting complex/cyclosome (APC/C). This ERK-dependent signature differs from the traditional KRAS signature and plays a crucial role in resistance to KRAS-ERK MAPK-targeted therapies in PDAC [17]. Therefore, developing novel strategies to overcome the failure of cancer immunotherapy is therefore of paramount importance, as the intricate and adaptive mechanisms employed by tumor cells to evade immune detection remain a significant obstacle to achieving sustained therapeutic success.

## 3 Advantages of nanosystems for prodrug delivery

**Fig. 1:**
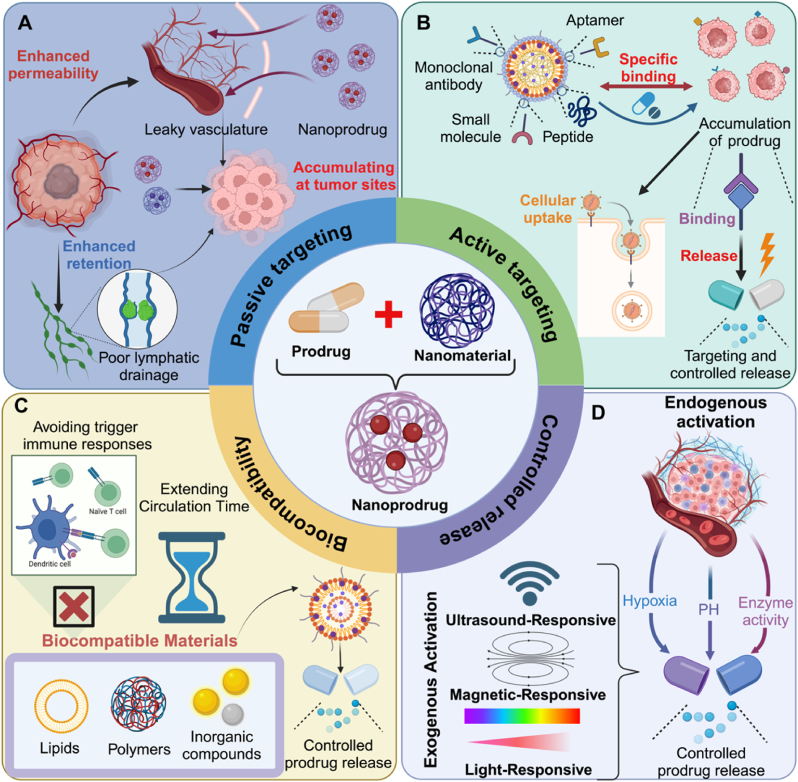
**Nanoparticle prodrug systems offer several advantages in cancer immunotherapy.** (A) Passive targeting via the enhanced permeability and retention (EPR) effect, leveraging leaky tumor vasculature and poor lymphatic drainage for nanoprodrug accumulation(B) Active targeting using ligands (e.g., aptamers, monoclonal antibodies, peptides). for tumor-specific binding and cellular uptake.(C) Biocompatible materials (polymers, lipids, inorganic compounds) enabling controlled prodrug release, extended circulation time, and reduced immune recognition.(D) Stimuli-responsive activation: Endogenous triggers (hypoxia, pH, enzymes) and exogenous stimuli (ultrasound, magnetic fields, light) spatiotemporally control drug release.

Prodrugs are an effective strategy to improve the delivery and activation of pharmaceutical agents, with a focus on enhancing solubility, stability, and bioavailability (Table 1). Designed to remain pharmacologically inactive until metabolically converted into their active forms after administration, these modifications optimize therapeutic outcomes while minimizing the adverse effects associated with direct use of active drugs [18]. However, traditional prodrugs, despite their ability to improve solubility and bioavailability, often encounter limitations in targeted delivery and controlled release. Their systemic distribution can result in non-specific accumulation, leading to off-target effects and toxicity. Additionally, the reliance on in vivo metabolic activation introduces variability in therapeutic response, influenced by individual metabolic profiles. These challenges highlight the need for more advanced drug delivery approaches, such as nano-prodrugs, which offer precise targeting, controlled release, and improved therapeutic efficacy (Fig. 1) [19].

**Table 1:** Comparison of different types of nanocarriers for prodrug delivery.

Type	Material Examples	Targeting Mechanisms	Stimuli-Responsive	Advantages	Limitations
**Inorganic Nanocarriers**	AuNPs IONPs Silica nanoparticles	EPR effect Active targeting	Radiation pH Magnetic GSH	High stability Multifunctionality Strong targeting specificity	Potential long-term toxicity Slow metabolic clearance Complex functionalization processes
**Lipid-Based Organic Nanocarriers**	Liposomes Micelles Lipid-drug conjugates	Cell membrane fusion Receptor-mediated endocytosis	pH ROS Photothermal	High biocompatibility Loading both hydrophilic/hydrophobic drugs Easy functionalization	Poor stability Difficulty in controlled release
**Synthetic Polymer-Based Carriers**	PEG/PLGA PHDT Polyamidoamine dendrimers	Receptor targeting	GSH Light/temperature Enzymes	Multi-stimuli responsiveness High drug-loading capacity Controllable degradation	Polymer residues may cause inflammation Challenges in large-scale production
**Natural Polymer-Based Carriers**	Hyaluronic acid (HA) nanoparticles Chitosan Albumin nanoparticles	Receptor targeting Macrophage-dependent targeting	Enzymes pH ROS	High biosafety Natural targeting capability Easy modification	Batch-to-batch variability Limited drug-loading capacity
**Organic-Inorganic Hybrid Carriers**	MOF-gold nanoparticle composites Silica-based composites	Dual targeting (ligand + EPR) TME-responsive integration	Light + enzymes pH + GSH ROS + radiation	Synergistic targeting/multi-stimuli responses Enhanced drug stability Multifunctional integration	Complex synthesis Risk of uncontrollable biodistribution
**Carrier-Free Nanoplatforms**	Prodrug self-assembled nanoparticles (e.g., Pt prodrug-PEG conjugates) Protein-drug complexes (e.g., albumin)	Prodrug self-assembly targeting Reliance on TME features (e.g., high GSH)	Carrier-free activation Endogenous triggers (pH, enzymes, metabolites)	Simplified preparation High drug-loading efficiency Reduced immunogenicity	Over-reliance on targeting mechanisms Risk of nonspecific release Difficult regulation

**Table 2:** Overview of PEG-Based nanocarriers in prodrug delivery systems.

Nanocarrier System	Drug	Nanocarrier	Stimulation Mechanism	Disease	Immune Mechanism	Ref.
PEG2k-Fmoc-NLG	NLG919 PTX	PEG-2000	Enzymatic cleavage	Breast cancer, melanoma	Enhancing T-cell responses; Inducing ICD via PTX; Inhibiting IDO	(124)
PA NPs	PTX	Diacylphosphatidylethanolamine-PEG2000	Cysteine depletion-triggered release	Malignant cancer	Inducing ferroptosis and ICD; Activating dendritic cells and T cells	(51)
PTX@PoxMTP	PTX	PEGMA	ROS-responsive peroxalate decomposition	Solid tumors	Accelerates PTX release; induces ICD; reduces Tregs/M2-TAMs; boosts effector T-cell infiltration.	(52)
DOX/IND@NPs	DOX	PEG-PVA copolymer	pH Temperature	Breast cancer	Inducing ICD and CD8^+^ T-cell infiltration Inhibiting IDO pathway	(125)
PEG/DPPA-MMP-DOX	DOX ^D^PPA	PEG	MMP-dependent transcytosis and tumor penetration	Solid tumors	Enhancing CTL infiltration Reducing Tregs Inducing immune memory	(126)
GPS Nanosystem	GEM	PEG-PLA	Enzymatic cleavage PD-L1 upregulation	ICB-refractory tumors	Activating STING pathway Enhancing anti-PD-L1 targeting	(54)
NP-Pt-IDOi	Pt (IV)-C12	mPEG-PDLLA matrix	ROS	Osteosarcoma	Inducing cGAS-STING activation Enhancing T-cell activity	(55)
BLZ@S-NP/Pt	Pt (IV) BLZ-945	Thioketal core with mPEG layers	660 nm light	Solid tumors	Depleting TAMs Enhancing CTL activity	(56)
P1@Pt (IV)-C16@IR1061	Pt (IV)-C16 IR1061	mPEG5k-OH	NIR-II light	TNBC	Amplifying ICD and Pt-DNA crosslinking Enhancing CTL infiltration.	(127)
PBOXA@TQTCD	PBOXA	PBMA	NIR-II light	CRC	Promoting cytotoxic T lymphocyte infiltration	(57)
ROS-sensitive TK-DC/Cis (IV)	Cisplatin (IV)	mPEG5k-OH	ROS	NSCLC	Dendritic cell maturation CD8^+^ T/NK cell activation	(58)
IDOi-TFP	Perylene-caged chlorambucil	PSMA-OAm-PEG	Far-red light-driven triplet fusion photolysis	Colorectal cancer	Reversing T-cell suppression	(59)
MAL-NPs	R848-N3	MAL-PEG-PDLLA mPEG-PDLLA	Radiotherapy	CRC	Upregulating CD80/MHC-II Priming CD8^+^ T cells	(60)
BLPNs	Ppa NLG919	mPEG-GG-b-poly	PH GSH	CRC	Inducing ICD to promote CRT exposure Reducing kynurenine levels Suppressing Tregs	(128)

Nanoprodrugs have emerged as a next-generation therapeutic platform that integrates drug delivery and controlled activation within a single molecular architecture. Unlike conventional nanocarriers that passively encapsulate or adsorb unmodified drugs, nanoprodrugs are created through covalent modification of active pharmaceutical ingredients, enabling self-assembly into nanostructures with precise physicochemical properties. This carrier-free design offers multiple advantages, including higher drug loading, improved stability, reduced premature release, and stimuli-responsive activation. These systems are often engineered to leverage the enhanced permeability and retention (EPR) effect, promoting passive accumulation at tumor sites due to abnormal vasculature and impaired lymphatic drainage. In parallel, lymph node–targeted nanovaccines have been developed by tuning particle size, shape, surface charge, and ligand functionalization to improve homing to tumor-draining lymph nodes (TDLNs), which is essential for antigen-presenting cell activation and T cell priming. By combining spatiotemporal control of drug activation with immune-responsive targeting, nanoprodrugs represent a powerful strategy to enhance therapeutic efficacy and elicit durable antitumor immune responses [20,21] For instance, poly (ethylene glycol)-block-poly (D,L-lactic acid) (PEG-b-PLA) and poly (ethylene glycol)-block-poly (ε-caprolactone) (PEG-b-PCL) nano-assemblies enhance the solubility and delivery of anticancer prodrugs, such as acyl and oligo (lactic acid) ester taxane prodrugs. These nano-assemblies improve tumor targeting, reduce systemic toxicity, and increase plasma exposure, resulting in superior antitumor efficacy compared to conventional drug formulations [22].

Biocompatibility is another key advantage of nanomaterial-based systems. By using materials such as biocompatible lipids, polymers (e.g., poly (lactic-co-glycolic acid) (PLGA) or PEG), or inorganic compounds like silica, gold, and iron oxide, these nanoparticles are engineered to avoid triggering immune responses, thereby extending circulation time and ensuring safety during prolonged treatment. Nanoparticles can be engineered to provide controlled and sustained release of prodrugs, overcoming the limitations associated with traditional drug release kinetics. These systems can be responsive to environmental stimuli such as pH, temperature, redox potential, or enzymatic activity [23]. For instance, stimuli-responsive hybrid nanocarriers, composed of drug-loaded UiO-66 NMOFs and DNA tetrahedra gates, enable controlled release triggered by acidic pH or specific miRNAs. These carriers enhance cell permeation and selectively target malignant cells, demonstrating efficient drug delivery and cytotoxicity in tumor-specific conditions. This ensures localized activation of the prodrug, protecting healthy cells from unnecessary drug exposure and reducing systemic toxicity [24]. Additionally, the encapsulation of prodrugs in nanoparticles can also shield them from premature degradation, extending their circulation time and enhancing their therapeutic potential [25].

Active targeting is another powerful strategy employed in nanoparticle-based drug delivery systems. By functionalizing the surface of nanoparticles with targeting ligands such as monoclonal antibodies, peptides, aptamers, or small molecules that bind specifically to overexpressed receptors on tumor cells, the nanoparticles can be directed precisely to the tumor site, further improving therapeutic outcomes. This targeted delivery not only increases the accumulation of the prodrug at the tumor site but also facilitates cellular uptake via receptor-mediated endocytosis, resulting in higher intracellular drug concentrations and enhanced therapeutic efficacy [26]. A bone marrow-targeting nanosystem, CSF@E-Hn, uses haematopoietic stem cell-derived nanovesicles with gripper ligands (aPD-L1, aNKG2D) and colony-stimulating factor (CSF) to activate natural killer cells and target tumour cells. In mouse models of acute myeloid leukaemia and multiple myeloma, it promotes haematopoietic stem cell differentiation, enhances memory T cell generation, and prevents relapse, offering a potential therapeutic strategy for hematologic malignancies [27]. Likewise, poly (beta-amino-ester) (PBAE) nanoparticles were engineered to deliver a CpG-free plasmid encoding mutant herpes simplex virus type 1 sr39 thymidine kinase (sr39) to hepatocellular carcinoma (HCC) cells. Targeted delivery was achieved through a human alpha fetoprotein (AFP)-promoter, enabling selective expression and subsequent cancer cell killing upon activation with the prodrug ganciclovir [28]. Furthermore, nanoparticles can be designed to release their cargos upon binding to specific receptors, providing a combination of targeting and controlled release that enhances both the specificity and the potency of the prodrug. Cetuximab-conjugated PLGA nanoparticles (INPs) loaded with the paclitaxel palmitate (PCPL) prodrug target EGFR overexpressing lung cancer cells. These immunonanoparticles demonstrate enhanced cellular internalization, cytotoxicity, and significant tumor growth inhibition in vivo. The prodrug, encapsulated within the nanoparticles, forms a drug reservoir with sustained release of paclitaxel, improving therapeutic efficacy and survival in metastatic lung cancer models [29].

The activation mechanism of nanoparticle-based prodrugs can be categorized into two primary types: endogenous activation, which relies on intrinsic biological conditions, and exogenous activation, which utilizes external stimuli. Endogenous activation of nanoparticle prodrugs takes advantage of the unique physiological environments within diseased tissues, such as tumors, or specific biochemical pathways [30]. For instance, ahybrid nanomedicine combining a porphyrin MOF and AuNPs enhances tumor-targeted delivery and stability of the hypoxia-activated prodrug TPZ. The nanomedicine releases TPZ in response to intracellular phosphate, exacerbating tumor hypoxia during PDT. This synergistic approach activates chemotherapy under hypoxic conditions, significantly improving antitumor efficacy and metastasis inhibition [5]. Exogenous activation methods utilize external physical stimuli to trigger the release of the drug from the nanoparticle. Photothermal and photodynamic therapies are examples of light-responsive prodrugs, where nanoparticles are engineered to absorb light and convert it into heat or ROS to activate the prodrug in a targeted manner. Similarly, magnetic-responsive nanoparticles can be guided to the tumor site by an external magnetic field, with drug release triggered by changes in temperature or magnetic field. Ultrasound-responsive systems utilize sound waves to enhance the permeability of the nanoparticle carrier, promoting drug release upon exposure to ultrasonic waves [31]. For instance, PtIV-functionalized polyacrylate-based nanoparticles, activated by near-infrared (NIR) light at 808 nm, enable multimodal cancer treatment. These nanoparticles, composed of PtIV complexes, photosensitizers, and targeting peptides, are selectively reduced to PtII in tumor cells upon irradiation, releasing therapeutic agents. The nanoparticles accumulate in tumor tissues, effectively eradicating triple-negative breast cancer in a mouse model [32]. In addition, visible-light-triggered prodrug nanoparticles (LT-NPs) combine verteporfin, a cathepsin B-cleavable peptide, and doxorubicin (DOX) for tumor-specific delivery and immunogenic cell death. Upon visible light irradiation, LT-NPs release therapeutic agents, inducing cytotoxicity and stimulating dendritic cell maturation to enhance T-cell activation. The integration of multiple stimuli-responsive mechanisms, referred to as multimodal or dual-responsive systems, further enhances the specificity and control over drug release [33]. These systems combine two or more external or internal stimuli to trigger the prodrug activation, offering more precise control over the therapeutic process and reducing the potential for side effects. For instance, a dual-activated prodrug, BTC, combines a glutathione-responsive BODIPY photosensitizer and an ROS-sensitive thioketal linker to release camptothecin (CPT) in tumor cells. Encapsulated in amphiphilic nanoparticles, BTC leverages high GSH concentrations in tumors for targeted activation. Upon light exposure, ROS generated by BODIPY induces apoptosis while releasing CPT, showing effective antitumor activity in breast cancer models [34].

## 4 Nano-prodrugs for enhancing cancer immunotherapy

### 4.1 Inorganic nanocarriers for prodrug activation and enhancing cancer immunotherapy

**Fig. 2:**
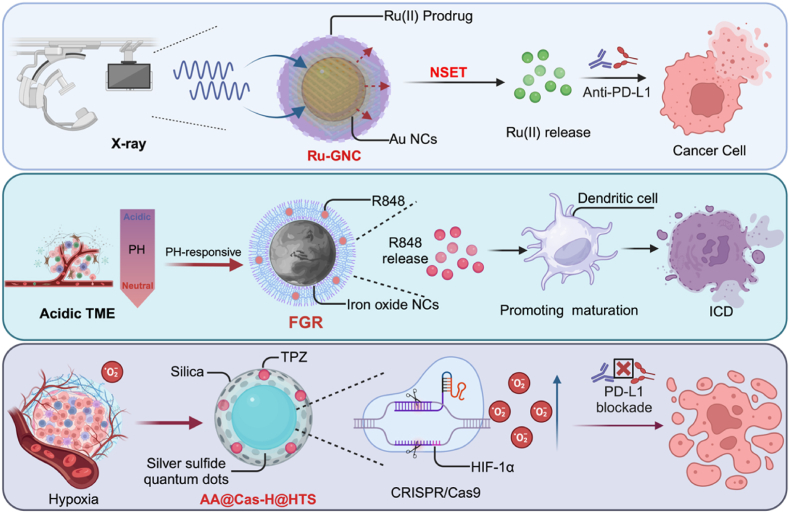
**Inorganic NC-based cancer therapy strategies**. Ru(II) Prodrug: X-ray activates Ru-GNC via NSET, releasing Ru(II). Combined with Anti-PD-L1, induces ICD in cancer cells.Acidic TME: pH-responsive FGR (Fe^3+^/R848 NCs) releases R848 in acidic TME, promoting DC maturation and ICD.Hypoxia: Ag_2_S QDs deliver AA@Cas-H@HTS (CRISPR/Cas9) to inhibit HIF-1α/PD-L1, alleviating hypoxia and enhancing T-cell immunity.

Inorganic nanocarriers offer significant advantages for prodrug delivery owing to their structural stability, high surface area, and tunable surface properties. These materials can be readily functionalized for targeted delivery and controlled drug release. Moreover, their intrinsic optical, magnetic, and electrical characteristics enable external activation—such as through radiation or pH shifts in the TME—allowing precise prodrug activation. These features make them valuable platforms for improving prodrug stability, circulation time, and therapeutic efficacy in cancer treatment [35]. (Fig. 2). Nanoprodrugs have garnered increasing attention for their capacity to induce ICD, thereby initiating a cascade of immune-mediated processes that are critical for the development of effective antitumor immunity. ICD is characterized by the release of TAAs and damage-associated molecular patterns (DAMPs), including calreticulin, ATP, and HMGB1, which function collectively to recruit and activate antigen-presenting cells (APCs), particularly DCs. This activation promotes DC maturation and facilitates efficient antigen cross-presentation, ultimately leading to the priming and expansion of CTLs. For example, MXene-based photothermal nanoplatforms (MXP) have been shown to induce ICD while co-delivering exogenous antigens and immunostimulatory adjuvants, thereby enhancing DC-mediated immune responses through both endogenous and exogenous pathways [36]. In addition,a radiation-triggered nanosurface energy transfer (NSET) strategy enables the controlled activation of metal prodrugs: a core–shell nanoplatform composed of gold nanoclusters and ruthenium-based hybrid coatings releases Ru(II) complexes upon X-ray irradiation, preserving reactive species in the TME and, when combined with PD-L1 blockade, induces immunogenic cell death (ICD) and robust antitumor responses [37]. In a different approach, gold nanoparticle-based bioorthogonal nanozymes convert an inactive imiquimod prodrug into its active form, selectively reprogramming macrophages from an anti-inflammatory to a pro-inflammatory phenotype, thereby enhancing phagocytosis and tumor cell killing without triggering systemic inflammation typically associated with imiquimod [38]. Furthermore, an iron oxide nanoparticle prodrug (FGR) with pH-sensitive properties releases the TLR7/8 agonist R848 under acidic TME conditions, promoting dendritic cell maturation and ICD while synergizing photothermal and chemodynamic effects to increase CD8^+^ T cell infiltration and suppress tumor growth and metastasis [39]. In addition, a TME-activated cascade nanoplatform (AA@Cas-H@HTS), constructed from silica-coated silver sulfide quantum dots, delivers CRISPR/Cas9 for HIF-1α knockdown and simultaneously activates the hypoxia-responsive prodrug TPZ, thereby alleviating hypoxia, disrupting PD-1/PD-L1 signaling, and enhancing T cell-mediated antitumor immunity [40]. Similarly, a layered double hydroxide (LDH)-based nanohybrid co-delivering a cisplatin prodrug and an immune checkpoint inhibitor enhances immunochemotherapy by increasing cisplatin accumulation, DNA binding, and apoptosis, while suppressing indoleamine-2,3-dioxygenase to restore T cell activity and promote tumor cell recognition [41]. Moreover, a Co(III) prodrug (Co2) encapsulated in glutathione-sensitive polymeric nanoparticles (NP-Co2) induces localized cytotoxicity and type II ICD by activating the GRP78/p-PERK/p-eIF2α/CHOP pathway, and works synergistically with αPD-1 therapy to augment both chemotherapy and immune checkpoint blockade [42]. Finally, acid-sensitive nanoscale coordination polymer particles (NCPs), formed from zinc ions (Zn^2+^) and phosphate groups, co-deliver a carboplatin prodrug, PD-L1-targeting siRNA, and digitoxin, triggering apoptosis, enhancing immunogenicity, and downregulating PD-L1 to reactivate antitumor immune responses and suppress tumor progression and metastasis [43]. Recent advances in inorganic nanocarriers have demonstrated that, beyond chemical composition, mechanical properties also play a pivotal role in modulating prodrug activation and immune responses. For example, soft mesoporous organosilica-based nanovaccines (SMONVs) with tunable elasticity have been shown to enhance DC uptake and promote endosomal escape, thereby improving antigen cross-presentation and eliciting robust CD8^+^ T cell-mediated antitumor immunity. This elasticity-dependent behavior also facilitates lymphatic trafficking and contributes to the development of durable immune memory. Similarly, TME-responsive inorganic nanoplatforms, such as the transformable trifecta nanovaccine (TriNV), exploit softening-induced enhancement of TAA uptake and ICD-mediated antigen release, while simultaneously remodeling the TME through Mn^2+^-mediated STING activation and hypoxia alleviation. These examples underscore the potential of mechanically adaptive inorganic nanomaterials not only to improve prodrug delivery efficiency but also to synergistically modulate the immune landscape, offering promising avenues for integrated chemoimmunotherapy.

Inorganic nanocarriers offer structural precision and stimulus-responsiveness not easily achieved with organic systems, enabling externally controlled prodrug activation in the tumor microenvironment. Noble metal-based platforms (e.g., Au, Ru) support highly specific activation but suffer from poor degradability and potential long-term accumulation. Transition metal-based carriers (e.g., Fe, Co, Zn) provide redox or pH sensitivity suitable for TME-adaptive release, yet require tight dose control to avoid cytotoxicity. Importantly, the immunological behavior of inorganic materials remains poorly defined, with unpredictable innate immune activation and rapid clearance unless surface-modified. Future designs should aim to integrate catalytic functionality with tunable immune interactions and degradability for safer, more effective immunotherapy.

### 4.2 Organic nanocarriers for prodrug activation and enhancing cancer immunotherapy

#### 4.2.1 Lipid-based nanocarriers

**Fig. 3:**
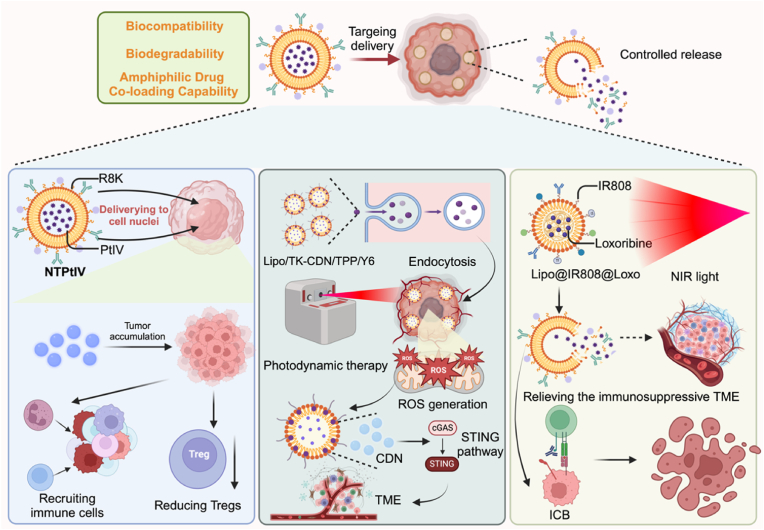
**Lipid-based nanocarriers for enhanced cancer therapy and immune modulation.** LNPs and liposomes, characterized by biocompatibility, biodegradability, and the ability to co-load both hydrophobic and hydrophilic drugs, are utilized for targeted drug delivery in cancer therapy. NTPtIV, a platinum (IV) prodrug conjugated with a nucleus-targeting peptide (R8K), is self-assembled into lipid nanoparticles for enhanced drug delivery to cancer cell nuclei, improving drug uptake and overcoming resistance. In vivo, NTPtIV accumulates in tumors, recruits immune cells (CD4^+^ and CD8^+^ T cells), and reduces Tregs. Lipo/TK-CDN/TPP/Y6, a nanosystem integrating PDT, PTT, and immunotherapy, facilitates ROS generation and activates the STING pathway under laser irradiation, promoting immune cell infiltration into the TME and amplifying therapeutic efficacy. Lipo@IR808@Loxo combines photothermal agent IR808 and TLR7 agonist loxoribine for NIR light-triggered therapy, activating antigen-presenting cells and relieving the immunosuppressive TME, leading to enhanced T-cell responses and the abscopal effect when combined with ICB.

Lipid-based nanocarriers, such as lipid nanoparticles (LNPs) and liposomes, are ideal for prodrug delivery due to their biocompatibility, biodegradability, and ability to encapsulate both hydrophobic and hydrophilic drugs. Their lipid bilayer structure stabilizes prodrugs, preventing premature degradation, while enabling controlled release. Additionally, they can be easily functionalized for targeted delivery and have the ability to fuse with cell membranes, promoting efficient cellular uptake. These characteristics make lipid-based nanocarriers highly effective for enhancing the stability and therapeutic potential of prodrugs in cancer therapy (Fig. 3) [44,45]. A Pt (IV) prodrug (C16-OPtIV-R8K) was designed by conjugating a hydrophobic lipid with a nucleus-targeting peptide, enabling self-assembly into uniform lipid nanoparticles (NTPtIV). NTPtIV enhances drug delivery to cancer cell nuclei, improving drug uptake and overcoming resistance. In vivo, NTPtIV exhibits efficient tumor accumulation, suppresses growth, and recruits immune cells, including CD4^+^ and CD8^+^ T cells, while reducing Tregs [46]. In addition, lipo/TK-CDN/TPP/Y6, a cyclic dinucleotide-based nanosystem, integrates PTT, photodynamic therapy, and immunotherapy for melanoma treatment. The mitochondria-targeting liposomes encapsulate a ROS-responsive prodrug and photoresponsive agents, facilitating enhanced cellular uptake and ROS generation under laser irradiation, leading to effective tumor inhibition. Beyond its direct cytotoxic effects, the nanosystem activates the STING pathway, triggering robust anti-tumor immune responses. This activation promotes the infiltration of immune cells into the TME, further amplifying the therapeutic efficacy [47]. Likewise, a dual-functional liposome (Lipo@IR808@Loxo) integrates a photothermal agent (IR808) and TLR7 agonist prodrug (loxoribine) for NIR light-triggered therapy. NIR irradiation enhances PTT to kill tumor cells, release tumor antigens, and activate antigen-presenting cells. The co-delivered loxoribine relieves the immunosuppressive TME, inducing a potent T-cell response. Combined with ICB, this nanoplatform promotes primary tumor elimination and inhibits distant tumor growth, enhancing the abscopal effect [48].

### 4.3 Polymer-based nanocarriers

Polymer-based nanocarriers are widely used for prodrug delivery due to their biodegradability, biocompatibility, and ease of functionalization for targeted delivery. These carriers can encapsulate both hydrophobic and hydrophilic prodrugs, protecting them from premature degradation and enabling controlled release. The structural diversity of polymers allows for precise control over particle size, surface charge, and drug release profiles, optimizing therapeutic efficacy. Polymer-based nanocarriers are classified into two categories: synthetic polymers like PLGA and PEG, which offer precise control, and natural polymers such as chitosan, alginate, and albumin, known for their biocompatibility. These properties make polymer-based nanocarriers ideal for enhancing the stability and therapeutic potential of prodrugs, particularly in cancer therapy [49,50].

#### 4.3.1 PEG-based nanocarriers

**Fig. 4:**
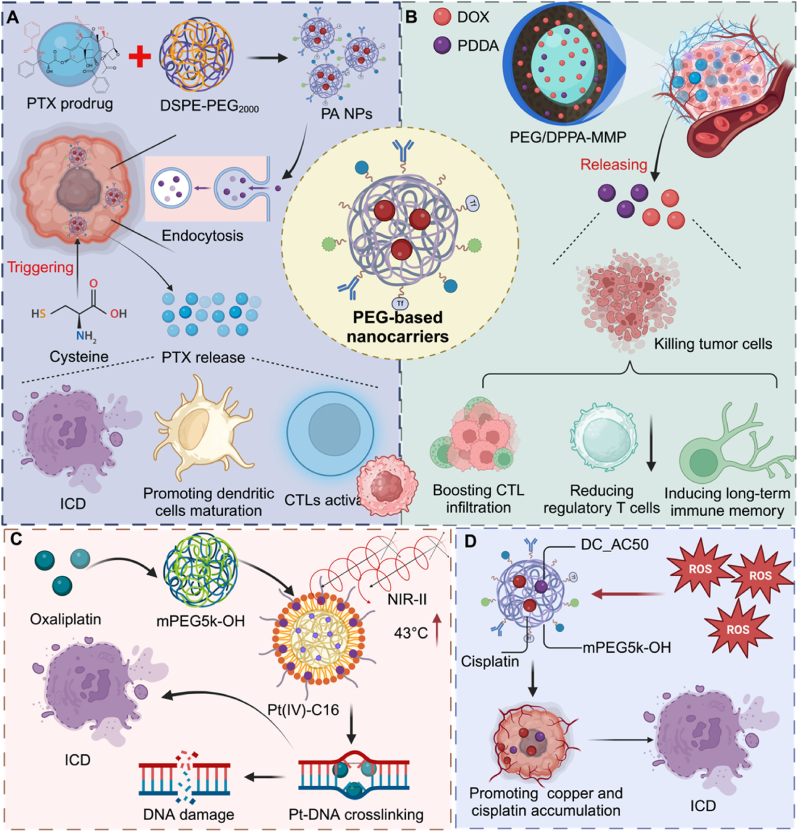
**Polymer-based nanocarriers for enhanced prodrug delivery and cancer immunotherapy.** Polymer-based nanocarriers, including PEG-based nanoparticles, are utilized for the delivery of prodrugs, enhancing both chemotherapy and immunotherapy. (A) PTX prodrug-loaded PEG-based nanocarriers (PA NPs) trigger PTX release via cysteine depletion, inducing oxidative stress, ferroptosis, and immunogenic cell death (ICD). This promotes dendritic cell maturation and T cell activation, boosting antitumor immunity. (B) DOX and PDDA-loaded PEG/DPPA-MMP nanoparticles enhance tumor accumulation and release DOX and PDPA in the TME, killing tumor cells, boosting CTL infiltration, and inducing long-term immune memory. (C) Oxaliplatin-based prodrug nanoparticles, encapsulated in mPEG5k-OH, utilize NIR-II PTT to enhance DNA damage and ICD, amplifying tumor cell killing and immune activation. (D) Copper and cisplatin-loaded mPEG5k-OH nanoparticles, incorporating DC_AC50, inhibit copper-ATPase pathways, promoting ROS production and inducing ICD, thereby enhancing immune responses and therapeutic efficacy.

PEG-based nanocarriers have gained prominence in prodrug delivery for cancer therapy due to their biocompatibility, prolonged circulation, and structural versatility (Fig. 4). For instance, a PTX prodrug formulated with diacylphosphatidylethanolamine-PEG2000 selectively releases PTX under cysteine depletion, triggering ferroptosis and ICD to activate dendritic cells and enhance T cell responses while minimizing systemic toxicity. Similarly, a ROS-responsive PEG-based carrier co-delivering PTX and 1-MT accelerates PTX release and induces ICD, while reducing Tregs and M2-TAMs, thereby promoting effector T cell infiltration and tumor clearance [51]. Another TME-activable PEG/DPPA-MMP nanoparticle co-delivers DOX and a PD-L1 antagonist, enhancing tumor accumulation and penetration, and subsequently increasing CTL infiltration, reducing Tregs, and establishing long-term immune memory [52]. In another study, a carrier-free nanoprodrug system (PEG@D:siRNA), formed via π–π stacking and electrostatic interactions between a DOX prodrug and PD-L1 siRNA, not only triggered ICD but also effectively downregulated PD-L1 expression in tumor cells. This dual-functional platform further augmented DC maturation and enhanced CTL infiltration within the tumor microenvironment [53].

In a related design, a PEG-PLA nanoplatform encapsulating gemcitabine, a STING agonist, and anti-PD-L1 antibody enhances chemoimmunotherapy by increasing drug accumulation and activating CD8^+^ T cells in ICB-resistant tumors [54]. Composite mPEG-PDLLA nanoparticles (NP-Pt-IDOi) co-loaded with a Pt (IV) prodrug and IDO inhibitor NLG919 promote DNA damage, activate the cGAS-STING pathway, and inhibit tryptophan metabolism, thus amplifying CD8^+^ T cell responses [55]. A nanocarrier featuring a thioketal core and PEGylated shell (BLZ@S-NP/Pt), co-delivering Pt (IV) and BLZ-945, undergoes size shrinkage under light to enhance tumor penetration and selectively deplete TAMs, improving therapeutic efficacy [56].

Furthermore, a PBMA-based PEG nanoparticle carrying an oxaliplatin prodrug and NIR-II fluorophore enables image-guided chemo-photothermal therapy, enhances intratumoral drug accumulation, and promotes ICD and CTL infiltration in colorectal cancer [57]. A ROS-sensitive mPEG5k-OH nanoparticle co-loaded with a copper chaperone inhibitor and cisplatin (IV) increases intracellular copper accumulation and ROS generation via Atox1-ATPase pathway inhibition, inducing ER stress and sustained immune activation [58]. A metal-free system using a BODIPY/perylene combination encapsulated in PSMA-OAm-PEG nanoparticles enables far-red light-triggered prodrug activation, while co-delivery of an IDO inhibitor enhances systemic antitumor immunity with minimal phototoxicity [59]. Besides chemotherapeutic payloads, PEG-based nanocarriers can also deliver immune-modulatory prodrugs. For example, maleimide-modified PEG-PDLLA nanoparticles carrying a resiquimod prodrug improve ICI therapy by promoting antigen uptake and facilitating radiotherapy-induced activation. When combined with anti-PD-1 and radiation, they enhance tumor accumulation and trigger robust antitumor responses [60].

PEG-based nanocarriers provide excellent circulation stability and reduced clearance, supporting their widespread use in prodrug delivery. However, their immunological inertness can impair interactions with antigen-presenting cells and limit endosomal escape, potentially weakening downstream immune responses. Moreover, repeated exposure may induce anti-PEG antibodies, and high molecular weight PEG chains may accumulate in vivo, raising concerns about long-term biocompatibility. To address these limitations, emerging designs incorporate cleavable or sheddable PEG shells and immune-responsive surface features, aiming to balance delivery stability with effective immune activation in the tumor microenvironment.

#### 4.3.2 PHDT

PHDT (Poly (HPMDA-disulfide-tert-Butyl (2,3-dihydroxypropyl) carbamate)-mPEG) is a multifunctional synthetic polymer incorporating reduction-responsive disulfide bonds, hydrophobic segments, and hydrophilic mPEG chains. This design enables PHDT to undergo structural changes or trigger drug release in reductive environments. For instance, a glutathione-responsive amphiphilic polymer (PHDT-Pt-In) delivers a Pt (IV) prodrug and COX-2 inhibitor indomethacin, overcoming chemoresistance in pancreatic cancer. The polymer self-assembles into nanoparticles (Pt-In NP) that release indomethacin upon disintegration in cancer cells, inhibiting COX-2 expression and enhancing platinum-induced pyroptosis. In vivo, Pt-In NP inhibits tumor growth, induces immune responses, and, when combined with anti-PD-L1 therapy, suppresses metastatic tumors, transforming cold tumors into hot ones [61]. In addition, a glutathione-responsive PHDT-OH based nanoparticle (PFS-NP) was developed, incorporating a cisplatin prodrug (Pt-OH), a disulfide bond-based polyphenol (PP-SS-DA), and iron ions (Fe3^+^). This configuration facilitates cellular uptake and glutathione depletion, disrupting redox balance and activating cisplatin. The subsequent reaction produces H2O2 and hydroxyl radicals via the Fenton reaction, triggering immunogenic cell death, enhancing dendritic cell maturation, and boosting antitumor activity [62].

#### 4.3.3 Other synthetic organic polymer-based nanocarriers

Beyond PEG and PHDT systems, a variety of synthetic organic polymer-based nanocarriers have been designed to deliver prodrugs and modulate antitumor immunity. A tumor-penetrating polyamidoamine (PAMAM)-based nanoparticle (SPN@Pro-Gem) enables size-shrinkage in acidic conditions, facilitating deep gemcitabine delivery in pancreatic tumors while reducing immunosuppressive cells and upregulating PD-L1, thereby enhancing CTL infiltration [63]. Dendritic polymer-conjugated resiquimod (R848) nanoparticles reprogram TAMs from M2 to M1 phenotype, enhancing antigen presentation and CD8^+^ T cell recruitment with minimal systemic toxicity [64]. A pseudo-conjugated polymer (PSP)-based Pt (IV) prodrug (PSP-Pt), co-assembled with lipid polymers, enables NIR-II light-triggered reduction and oxaliplatin release, combining photothermal and chemo-immunotherapy [65].

Other multifunctional designs include a cinnamaldehyde-based polymer prodrug (PDPCA) that enhances radiosensitization by depleting GSH and inducing ferroptosis; encapsulation of ATRA further amplifies ROS generation and T cell responses when combined with anti-PD-1 therapy [66]. A related system (PolyRA@Oxa-c16) co-delivers retinoic acid and Pt (IV), promoting T cell-mediated tumor clearance and immune memory when combined with PD-L1 blockade [67]. A sonodynamic polymeric platform (EIPS), incorporating a Cathepsin B-cleavable exosome inhibitor prodrug, suppresses tumor exosome release and metastasis upon TME-specific activation [68].

Smart semiconducting polymer systems such as SPNI, composed of NIR-absorbing semiconductors and a TLR7 agonist-conjugated polymer, respond to acidic TME and NIR irradiation to induce ICD and generate an in situ cancer vaccine [69]. Likewise, an oxaliplatin–phthalocyanine coordination polymer combined with an IDO1 inhibitor prodrug forms a laser- and GSH-activatable nanosystem that promotes deep tumor penetration and restores antitumor immunity [70]. A fluorinated prodrug nanoformulation (FEM@PFC) delivers oxygen and depletes GSH, reversing Foxp3^+^ Treg-mediated suppression and reshaping the immune microenvironment [71].

In addition, synthetic polymer systems have been integrated with lipid or PEG-based components for combinatorial delivery strategies. For example, a GSH-responsive camptothecin (CPT) prodrug encapsulated in PEGylated lipid nanoparticles improves bioavailability, prolongs circulation, and enhances CD8^+^ T cell infiltration by promoting dendritic cell activation [72]. Lipid-PEG hybrid systems, such as Podo-NP and CbP-NP, sequentially deliver podophyllotoxin and carboplatin prodrugs, regulating angiogenesis, tumor vasculature, and apoptosis; co-administration with a CD40 agonist reverses immunosuppression and boosts T cell-mediated responses [73]. A multifunctional PEGylated nanovesicle loaded with doxycycline enhances antigen presentation by inhibiting autophagy and increasing MHC-I, while its sheddable PEG shell and CRGDK ligand improve tumor targeting and circulation stability [74].

Further innovations include a STING-activating nanoadjuvant constructed by ionizable prodrug conjugation of a non-nucleotide agonist (MSA-2), which undergoes PEG deshielding in acidic TME to enable controlled release and potent STING activation in colorectal and triple-negative breast cancer models [75]. A ROS-responsive nanoparticle delivering CPT and Pt (IV) prodrugs promotes DNA damage and cGAS-STING signaling, stimulating dendritic cells and enhancing CD8^+^ T cell infiltration [76]. A BBB-penetrating nanotheranostic (T7-PEG5k-DSPE) co-delivering a JQ1 prodrug and NIR-II fluorophore enables targeted photo-immunotherapy in glioblastoma by suppressing PD-L1 and enhancing immunogenicity through photothermal effects [77]. Finally, a dual-responsive micellar system (PEG-PLL-DMMA/P(HCPT-DTDA)-PEI) co-delivering siTGF-β and hydroxycamptothecin undergoes size shrinkage and charge reversal in acidic TME, improving tumor penetration, reducing immune tolerance, and reprogramming the tumor microenvironment via EMT inhibition and TGF-β blockade [78].

Synthetic organic polymer-based nanocarriers provide tunable architectures and responsive functionalities for prodrug delivery and immune modulation. However, their non-natural chemical structures may lead to suboptimal interactions with immune cells, including limited recognition by antigen-presenting cells and poor lymphoid tissue trafficking. In some cases, the lack of inherent immunogenicity or bioactivity necessitates the addition of external immune stimulants, increasing formulation complexity. Moreover, certain synthetic backbones—such as pseudo-conjugated or highly hydrophobic polymers—may exhibit slow or incomplete biodegradation, potentially leading to intracellular accumulation or inflammatory responses. Careful material selection and surface engineering are essential to enhance immunological compatibility and ensure safe, efficient translation of synthetic polymer-based systems into immune-targeted cancer therapies.

#### 4.3.4 Natural organic polymer-based nanocarriers

**Fig. 5:**
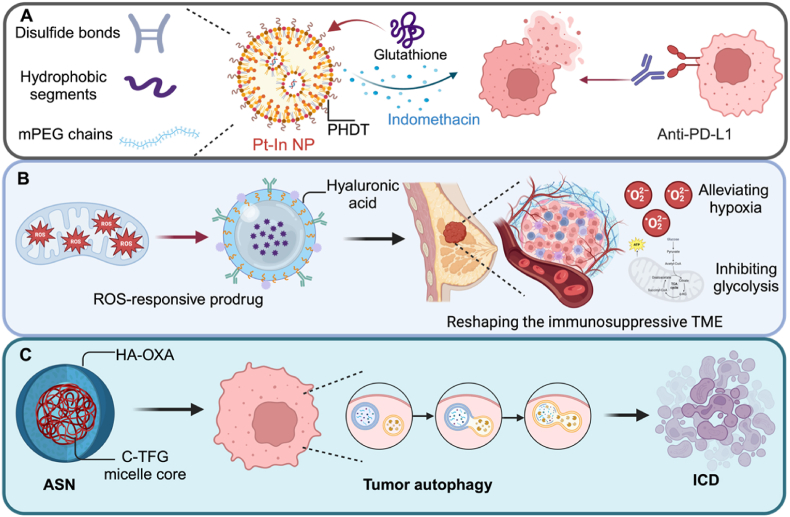
**Natural Polymer-Based Nanocarriers for Enhanced Tumor Immunotherapy.** (A) Pt-In NPs are engineered with disulfide bonds, hydrophobic segments, and mPEG chains for tumor-specific targeting. Glutathione triggers the release of indomethacin and PHDT, which promote anti-PD-L1 immune responses. (B) ROS-responsive prodrugs, like those containing hyaluronic acid, modulate oxidative stress to alleviate tumor hypoxia and inhibit glycolysis, reshaping the immunosuppressive (TME for enhanced chemotherapy and immunotherapy efficacy. (C) A hyaluronic acid-based core-shell nanoplatform (HA-OXA) promotes tumor autophagy and induces ICD, stimulating immune responses and enhancing treatment outcomes.

Natural polymer-based nanocarriers, such as those derived from hyaluronic acid, chondroitin sulfate, and albumin, provide a highly biocompatible platform for prodrug delivery, enabling targeted accumulation and controlled release in tumor tissues (Fig. 5). In triple-negative breast cancer (TNBC), ROS-responsive hyaluronic acid nanoparticles co-loaded with DOX and the BRD4 inhibitor JQ1 modulate oxidative stress and glycolytic activity. This system alleviates hypoxia, promotes DOX release, and inhibits glycolysis, thereby reshaping the immunosuppressive tumor microenvironment and enhancing chemo-immunotherapy efficacy [79]. Another system, HA-Psi-DOX, conjugates DOX to hyaluronic acid via an MMP-sensitive peptide, allowing for tumor-specific enzyme-mediated activation. DOX release induces PD-L1 upregulation, which is counteracted by co-administration with anti-PD-1 therapy, improving immune engagement and treatment outcomes [80].

A detachable core-shell platform (DOX@HA-MMP-2-DEAP/CXB) co-delivers DOX and celecoxib (CXB), releasing them in response to MMP-2 activity and acidic pH, respectively. DOX induces ICD, while CXB enhances dendritic cell and T cell infiltration and reduces immunosuppressive cells, effectively remodeling the TME [81]. In pancreatic cancer models, a supramolecular nanoplatform formed via host–guest interactions between cyclodextrin-modified HA and pyropheophorbide A–JQ1 heterodimers enables CD44-targeted photoimmunotherapy. Light-triggered ROS generation promotes ICD, while JQ1 suppresses PD-L1 and c-Myc to counteract immune evasion and glycolysis [82].

Biomimetic hyaluronidase-responsive nanoparticles (mCAuNCs@HA), co-loaded with pheophorbide A and a ROS-sensitive paclitaxel dimer, allow for size shrinkage and on-demand drug release. The inclusion of anti-PD-L1 peptides enhances cytotoxic T lymphocyte and NK cell activation under photodynamic and chemotherapeutic stress [83]. Chondroitin sulfate-based nanoparticles co-delivering Ce6 and retinoic acid (RA) achieve Golgi-targeted photodynamic therapy, disrupting cytokine secretion and promoting dendritic cell maturation when combined with CpG oligodeoxynucleotide as an immunoadjuvant, thus amplifying systemic antitumor responses [84] In a distinct approach, a Pt (IV) prodrug axially modified with perfluorocarbon chains is encapsulated into human serum albumin nanoparticles. This system targets tumors efficiently and triggers ROS production in the endoplasmic reticulum, inducing ICD and demonstrating potent antitumor activity in osteosarcoma models [85] (Fig. 5).

Natural polymer-based nanocarriers benefit from excellent biocompatibility and receptor-mediated targeting, but their biological origin also introduces immunological complexity. Variability in glycosylation, protein content, or residual bioactive motifs may lead to unpredictable interactions with immune receptors, potentially triggering innate immune responses or rapid clearance. Moreover, their soft and hydrated structure, while favorable for biocompatibility, often compromises mechanical stability and controlled drug release, especially under physiological shear or enzymatic degradation. For effective application in immunotherapy, future designs must carefully balance immune compatibility with delivery precision, ideally through partial chemical modification or hybridization strategies that preserve biological advantages while improving functional reliability.

#### 4.3.5 Synthetic/natural hybrid polymer-based nanocarriers

Hybrid nanocarriers combining synthetic and natural polymers offer a promising strategy to integrate structural versatility with biocompatibility for prodrug-based immunotherapy. One example is a pH-responsive nanoparticle system that co-delivers R848 and DOX for breast cancer treatment. R848 is encapsulated in poly (L-histidine) nanocores, while DOX is conjugated to hyaluronic acid to form HA-DOX prodrug nanoparticles. Upon TME acidification, R848 is released to activate immune cells, and DOX induces tumor cell cytotoxicity, enabling synchronized chemoimmunotherapy [86]. Similarly, HA-CDDP/PMet nanoparticles co-deliver cisplatin and metformin, achieving enhanced tumor accumulation and apoptosis while activating AMPK-α signaling and inhibiting mTOR, thereby strengthening antitumor immunity in lung cancer models [87]. A more advanced design, the autophagy-sensitive ASN platform, utilizes a HA-oxaliplatin prodrug shell and C-TFG micelle core. Upon cellular entry, it initiates mild autophagy and ICD, followed by amplified autophagy induction, boosting antigen presentation and enhancing immunotherapy efficacy in CT26 tumors [88]. Additionally, bispecific chitosan-based nanoparticles linking NLG919 and JQ1 through disulfide bonds are activated by glutathione within tumor cells. This dual-drug release restores T cell responses and, with a tumor-targeted, acidity-activated shell, enhances PDT-induced ICD, enabling combinatory immune modulation [89].

Hybrid polymer-based nanocarriers combine the chemical versatility of synthetic polymers with the biological compatibility of natural materials, offering a promising strategy for immune-targeted prodrug delivery. However, the inherent chemical disparity between the two components can result in interfacial instability, asynchronous degradation, or impaired drug release kinetics. In some systems, synthetic segments may hinder immune cell uptake or introduce persistent residues, while natural components may compromise structural integrity. Achieving a functionally balanced hybrid requires precise control over composition, degradation profiles, and immunological behavior, making material-level optimization essential for effective immunotherapeutic application.

### 4.4 Organic-inorganic hybrid nanocarrier

**Fig. 6:**
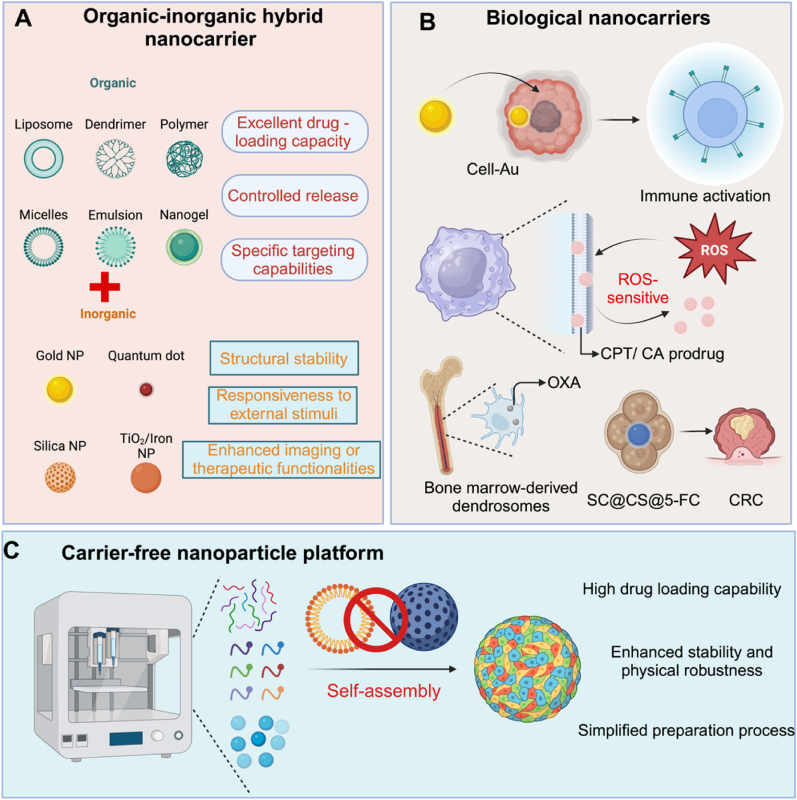
**Other Nanoparticle Prodrugs for Enhancing Cancer Immunotherapy** (A) Organic-inorganic hybrid nanocarriers combine organic materials (liposomes, dendrimers, polymers, micelles, emulsions, and nanogels) with inorganic components (gold nanoparticles, quantum dots, silica nanoparticles, and TiO_2_/iron nanoparticles), offering controlled drug release, improved drug loading, tumor-targeted delivery, and enhanced imaging or therapeutic functionalities. (B) Biological nanocarriers, such as cell-Au conjugates, bone marrow-derived dendrosomes, and SC@CS@5-FC, promote immune activation and enable ROS-sensitive drug delivery, significantly enhancing tumor-targeting capabilities and immunotherapy effectiveness. (C) Carrier-free nanoparticle platforms rely on self-assembly of drug molecules into nanoparticles, offering enhanced stability, high drug loading capacity, and a simplified preparation process, while maintaining the potential to modulate immune responses for more effective cancer treatment.

Organic-inorganic hybrid nanocarriers integrate the complementary advantages of biocompatible organic materials and structurally robust inorganic components, creating multifunctional platforms for precise prodrug delivery and immune modulation (Fig. 6) [90]. The organic fraction—typically composed of polymers or biomolecules—facilitates drug encapsulation, controlled release, and tumor-specific targeting. In parallel, inorganic elements such as metal nanoparticles or oxides contribute enhanced stability, responsiveness to external stimuli (e.g., ROS, light), and added therapeutic or imaging functionalities. This synergy enables precise control over prodrug activation and immune engagement within the tumor microenvironment.

One example involves a ferrocene- and thioketal-containing polymeric nanoplatform for Artoxplatin delivery, which releases the platinum agent in response to ROS. The resulting oxidative stress not only induces tumor cell death but also triggers ICD and promotes dendritic cell maturation, enhancing antitumor immunity [91]. Another platform combines copper-doped MOFs with hyaluronic acid to form a dual-responsive nanocarrier encapsulating a disulfiram prodrug (DQ). This system targets CD44^+^ tumor cells and responds to phosphate and hydrogen peroxide, releasing Cu(DTC)_2_ to induce apoptosis and ICD, thereby improving ICI response and systemic immunity [92].

Additionally, RPMANB nanoparticles co-deliver an IDO inhibitor (NLG919) and a chemotherapeutic prodrug within a MOF-based carrier. Collapse of the MOF under tumor-specific conditions releases NLG919 to suppress IDO-mediated immune tolerance, while NIR-triggered plasmonic catalysis activates the chemotherapeutic component, inducing ICD and amplifying antitumor responses [93]. A more integrated approach involves a dual-responsive hydrogel composed of PpIX-modified iron oxide nanoparticles (IONPs) and ROS-cleavable aPD-L1 prodrug nanoparticles. Upon in situ gelation and NIR irradiation, the system generates ROS, enabling photodynamic and chemodynamic therapy, while simultaneously releasing aPD-L1 to enhance immune checkpoint blockade efficacy [94].

Organic-inorganic hybrid nanocarriers harness the drug-loading versatility of organic materials and the structural robustness and stimulus-responsiveness of inorganic components, enabling precise prodrug activation and immune modulation. Their multifunctionality supports synergistic strategies such as photodynamic therapy, immune checkpoint blockade, and ICD induction. Nonetheless, the integration of chemically and physically distinct domains introduces critical challenges. In particular, mismatched degradation kinetics, inconsistent drug release, or destabilizing interfacial interactions can lead to premature cargo leakage or diminished therapeutic specificity. Moreover, the immunological consequences of inorganic surface exposure—such as unintended innate immune activation or clearance—remain insufficiently characterized.

### 4.5 Biological nanocarriers

Biological nanocarriers, derived from natural or bio-compatible materials, offer distinct advantages for drug delivery, particularly in enhancing bioavailability and targeting therapeutic agents to specific sites. These carriers, including biopolymers and cell-derived vesicles, exhibit excellent biocompatibility, low toxicity, and the ability to navigate complex biological environments. Their structural versatility allows for the efficient encapsulation and controlled release of drugs, while their inherent targeting properties improve selective drug delivery, minimizing off-target effects. As such, biological nanocarriers are increasingly recognized for their potential in overcoming the limitations of conventional drug delivery systems [95]. A tumor whole-cell catalytic vaccine (TWCV) is developed by incorporating gold ions into live cancer cells, enabling NIR-controlled catalytic activation. The engineered Cell-Au maintains tumor antigens and homing ability while providing prodrug-based 1O2 production in hypoxic conditions. This nano prodrug enhances dendritic cell activation and macrophage repolarization, inducing potent antitumor immune responses in 4T1 tumor-bearing mice [96]. Additionally, TAM membrane-camouflaged nanodecoys, incorporating a self-amplifying ROS-sensitive CPT/cinnamaldehyde (CA) prodrug, were developed for synergistic immunotherapy. The ROS-cleavable prodrug nanoparticle triggers a positive feedback loop of ROS-mediated drug release and ROS generation, inducing ICD. TAM targeting and depletion, combined with effector T cell infiltration, enhances antitumor immunity [97]. Interestingly, a redox-responsive nanoparticle (NP) platform was developed by fusing bone marrow-derived dendrosomes (mDs) with oxaliplatin (OXA) prodrugs to induce immunogenic tumor cell death and enhance immune response. The NP/mDs system potentiated antitumor immunity by promoting tumor antigen presentation, immune cell infiltration, and differentiation. In combination with PD-L1 blockade, this strategy improved tumor growth inhibition and metastasis suppression by reinforcing tumor-specific T cell responses [98]. Moreover, a fungi-triggered in situ chemotherapeutic generator (SC@CS@5-FC) utilizes Saccharomyces cerevisiae (SC) to target colorectal tumors and release the prodrug 5-fluorocytosine (5-FC) in response to hyaluronidase in the tumor microenvironment. The prodrug is converted to 5-fluorouracil (5-FU) by cytosine deaminase (CD), while SC and chitosan nanoparticles serve as immune adjuvants to enhance antitumor immunity [99].

Biological nanocarriers offer distinct advantages in biocompatibility, immune system integration, and active targeting through membrane proteins or endogenous signaling pathways. Their use of cell-derived membranes, immune vesicles, or microbial scaffolds enables simultaneous prodrug delivery and immune activation, often with minimal synthetic modification. These systems can enhance dendritic cell priming, antigen presentation, and cytotoxic T cell responses, making them promising tools in immunotherapy. However, the intrinsic complexity of biologically derived materials presents key challenges. Variability in membrane composition, surface protein density, and glycosylation patterns may lead to unpredictable immune recognition, resulting in rapid clearance by the mononuclear phagocyte system or unintended immune activation. Moreover, the biodistribution and pharmacokinetics of such carriers are difficult to standardize, and their interactions with host immune cells may differ depending on patient-specific factors (see Table 2).

### 4.6 Carrier-free nanoparticle platform

Carrier-free nanoparticle systems eliminate the need for inert delivery scaffolds by enabling the self-assembly of therapeutic components, thereby enhancing drug loading efficiency, stability, and targeted release (Table 3). Recent advances in self-delivering and carrier-free nanodrugs provide promising strategies to overcome limitations associated with conventional carrier-based systems. A notable example is the development of flexible biomimetic nanocapsules (OVAnano), which rely primarily on the antigen itself (ovalbumin) with minimal adjuvants to drive nanoassembly. These vesicle-like structures enhance DC uptake, facilitate endosomal escape, and robustly activate NF-κB signaling, thereby promoting efficient antigen presentation and eliciting strong CD4^+^ and CD8^+^ T cell responses. Another example is the carrier-free disulfiram-based nanodrug (CFDC), constructed via supramolecular self-assembly without the use of auxiliary carriers. By tailoring the morphology into nanodots, rods, or sheets, CFDC enhanced intracellular ROS generation and activated the JNK/p38 signaling axis, while simultaneously overcoming NF-κB–mediated resistance. This design led to improved induction of apoptosis and DNA damage in tumor models, highlighting the potential of morphology-controlled, self-assembling nanodrugs for efficient and mechanism-driven cancer therapy.For instance, a carrier-free platform co-delivering DOX, melittin (MPI), and anti-TOX siRNA induces ICD and reverses T cell exhaustion in liver cancer. DOX and MPI promote CD8^+^ T cell infiltration, while siTOX reduces exhaustion, collectively enhancing immune responses [100]. A glutathione/pH dual-responsive prodrug nanoparticle sequentially releases 5-aminolevulinic acid and DOX, promoting dendritic cell maturation, reducing immunosuppressive cells, and synergizing with anti-PD-1 therapy [101].

**Table 3:** Carrier-free nanoparticle platform.

Type	Compositions	Targeting Mechanisms	Effects	Clinical significance	Ref.
**FD/FM@siTOX NPs**	DOX, MPI, and siTOX	Enhancing ICD increase CD8^+^T-cell infiltration, mitigating CD8^+^ T cell exhaustion	Increasing CD8^+^ T cell infiltration	Producing a potent antitumor immune response in liver cancer and metastasis	(100)
**DSA NG**	Cross-linked nanogel, 5-aminolevulinic acid, DOX	Glutathione/pH dual-responsive	Leading to an excellent tumor-killing efficacy	Realizing synergistic anticancer therapy, serving as a real-time imaging probe of accurate cancer diagnosis.	(101)
**CAP-NPs**	DOX, FRRG	Selectively releasing cytotoxic DOX in cathepsin B-overexpressing cancer cells	Exhibiting high rate of complete tumor regression	Increasing clinical benefit by inducing an immune response preferentially only to targeted cancer cells	(102)
FU-SS-IND NPs	5-FU, IDO inhibitor	Disulfide linker-inducing GSH exhaustion, IND inhibiting GSH biosynthesis	Enhancing effector function of T cells for turning a "cold" tumor to a "hot" one	Demonstrating translatable potential for reversing drug resistance and enhancing immunotherapy of HCC	(104)
Mito-CMPN	Stimuli-responsive polyprodrugs	Inducing chemo-photodynamic therapy-caused mitochondrial stress in tumor cells	Reversing TIME in ovarian cancer subcutaneous model and high-grade serous ovarian cancer model	Demonstrating an effective strategy to improve therapeutic efficacy of immunosuppressive ovarian cancer	(105)
GEM-1MT	GEM, 1 MT	Triggering ICD, inducing selective MDSC depletion, inducing IDO inhibition in tumor cells and MDSCs.	Killing tumor cells, reversing TIME	Providing a new concept for rational design of a minimalist drug nanoplatform	(129)
Nap-CPT-HCQ-Yp	Phosphotyrosine motif, CPT, HCQ	Enhancing chemotherapy and autophagy inhibition, inducing ICD and activating T-cell responses	Achieving efficient inhibitions of primary tumors and distant tumors in breast tumor model	Offering a simple and feasible strategy for the design of "smart" multifunctional prodrugs	(113)
PD-NPs	Anti-PD-L1 peptide, cathepsin B-specific cleavable peptide, DOX	Taking up PD-NPs by PD-L1 receptor-mediated endocytosis, inducing ICD in cancer cells	Disrupting immune suppressing pathway, resulting in T cell proliferation and reinvigoration	Increasing the safety and efficacy of ICB in combination with chemotherapeutic agents	(108)
PARE NPs	Pyropheophorbide-a, R848	Ablating primary tumor directly, eliciting ICD, polarizes the M2-type TAM to M1-type TAM	Killing cancer cells, reversing "cold" and suppressive TIME	Demonstrating an effective strategy to enhance antitumor immunity enhanced	(109)

Cancer-activated prodrug nanoparticles (CAP-NPs) selectively release DOX in cathepsin B-overexpressing tumor cells, inducing ICD with reduced systemic toxicity. Stabilized by Pluronic F68, CAP-NPs minimize immune cell cytotoxicity while enhancing antitumor immunity [102]. Similarly, anti-PD-L1 peptide-conjugated DOX prodrug nanoparticles (PD-NPs) enable cathepsin B-triggered ICD and T cell activation through PD-L1 blockade [103]. A bioreducible nano-prodrug (FU-SS-IND NPs), linking 5-FU to an IDO inhibitor via disulfide bonds, depletes GSH and enhances T cell effector function, effectively converting cold tumors into hot ones [104].

Mitochondria-targeted polyprodrug nanoparticles (Mito-CMPN), loaded with cisplatin and mitoxantrone, induce ICD via mitochondrial stress and combine chemo-, photo-, and immunotherapeutic effects in ovarian cancer [105]. The multifunctional prodrug Nap-CPT-HCQ-Yp self-assembles into nanoparticles and transitions into nanofibers in response to tumor enzymes, releasing CPT and HCQ to synergistically promote ICD and T cell responses through autophagy inhibition [106]. Likewise, a CPT prodrug nanofiber enhances tumor penetration and overcomes T cell immune evasion by co-delivering PD-L1-silencing plasmids and hyaluronidase to degrade ECM and reduce PD-L1 expression. GSH-activated cisplatin prodrug nanoassemblies further promote intracellular aggregation and ROS generation, enhancing retention and ICD induction when combined with docetaxel and IR820 [107].

Beyond chemotherapeutics, carrier-free prodrug systems can deliver immune modulators or other small molecules to enhance immunotherapy. Tumor-activated PDNPs formed by self-assembling prodrugs improve pharmacokinetics and tumor targeting, enhancing ICD induction and immune checkpoint therapy responses [108]. A conjugated prodrug (PA-R848), composed of a photosensitizer and TLR7/8 agonist, self-assembles into esterase-responsive nanoparticles (PARE NPs), combining phototherapy-induced ICD with TAM repolarization to reverse immunosuppression [109]. A light-triggered PROTAC nanoassembly (LPN), targeting IDO, integrates photodynamic therapy and IDO degradation to simultaneously induce ICD and suppress Tregs [110]. Additionally, FeCO-IR820@FeIIITA nanoparticles co-deliver a thermosensitive CO prodrug and iron for synergistic photothermal, ferroptotic, and gas therapy. The system enhances tumor targeting via the Fenton reaction and, when combined with CTLA-4 blockade, suppresses tumor growth and metastasis while boosting antitumor immunity [111].

Carrier-free nanoparticle platforms eliminate traditional carrier matrices by utilizing the self-assembly of prodrug molecules themselves, offering high drug content and chemical definability. However, the absence of stabilizing scaffolds can compromise colloidal stability, leading to premature aggregation or disassembly in circulation. These systems often exhibit limited structural robustness under physiological conditions, and their pharmacokinetics are highly dependent on prodrug design, linker chemistry, and external stimuli. In the context of immunotherapy, effective immune activation requires not only controlled drug release but also retention within immune-relevant compartments—features that remain difficult to fine-tune in carrier-free formats. Addressing these challenges will be key to translating their formulation simplicity into therapeutic reliability.

## 5 Clinical translation of nanoprodrugs

With the rapid development of nanoprodrug technologies, several formulations have advanced from preclinical proof-of-concept to clinical evaluation, demonstrating safety, tolerability, and early signs of efficacy in human patients. These clinically investigated nanoprodrugs exemplify the translational potential of integrating drug delivery and controlled activation within a single nanostructured platform [112,113]. The following representative examples highlight the clinical performance of nanoprodrugs across various solid tumors, underscoring their promise in enhancing therapeutic outcomes while minimizing systemic toxicity.

Clinically evaluated nanoprodrugs represent a distinct class of therapeutics that integrate drug delivery and controlled activation within a single molecular framework, enabling targeted tumor accumulation and reduced systemic toxicity. K-912 (NC-6300) is a micelle-formulated epirubicin nanoprodrug designed to enhance tumor delivery while reducing systemic toxicity. In a first-in-human trial, K-912 was well tolerated up to 170 mg/m^2^, with minimal anthracycline-related adverse effects. One partial response and prolonged stable disease were observed among 19 patients with advanced solid tumors. Pharmacokinetics mirrored preclinical profiles [114]. In addition, NC-6300 is a pH-sensitive epirubicin nanoprodrug that accumulates selectively in tumors via the enhanced permeability and retention effect. In patients with advanced solid tumors, including soft-tissue sarcomas, NC-6300 was tolerated at doses up to 185 mg/m^2^, exceeding conventional epirubicin limits. Dose-limiting toxicities were manageable, and partial responses were noted in angiosarcoma and endometrial stromal sarcoma. Pharmacokinetics revealed dose-dependent epirubicin release [115]. CRLX101 is a nanoparticle-formulated camptothecin prodrug designed for sustained tumor delivery with reduced systemic toxicity. In recurrent ovarian cancer, CRLX101 showed a clinical benefit rate of 68 % as monotherapy, which increased to 95 % when combined with bevacizumab. The combination also improved progression-free survival with manageable toxicity. These results highlight the potential of nanoprodrugs to enhance the efficacy and tolerability of topoisomerase I inhibitors in platinum-resistant disease [116]. Notably, EP0057 is a nanoparticle-drug conjugate of camptothecin designed for sustained release and enhanced tumor retention. In combination with weekly paclitaxel, EP0057 achieved an objective response rate of 31.6 % in heavily pretreated ovarian cancer patients previously exposed to bevacizumab. The regimen was well tolerated, with manageable hematologic toxicity [117].

Collectively, these clinical studies validate the potential of nanoprodrugs to overcome the limitations of conventional chemotherapeutics by improving pharmacokinetic profiles, minimizing systemic toxicity, and achieving meaningful clinical responses in patients with advanced or refractory tumors. These findings also encourage the continued development of immunomodulatory nanoprodrugs designed to integrate immune activation with precise tumor targeting, paving the way toward clinical translation of nanomedicine-based cancer immunotherapy.

## 6 Challenges and perspectives

**Fig. 7:**
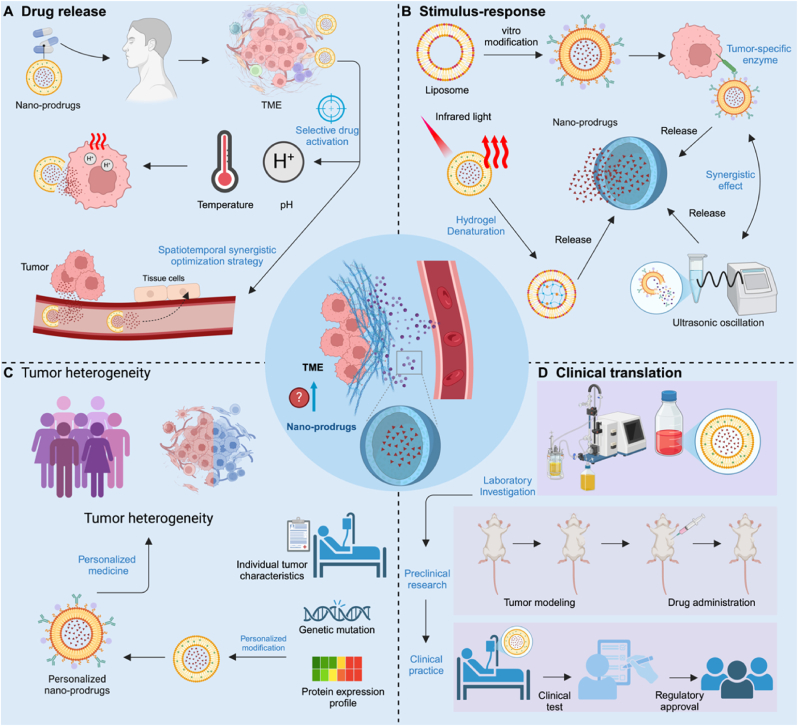
**Challenges and Strategies in the Development of Nano-Prodrugs for Cancer Therapy**. **(A)** The diagram illustrates the need for precise control of drug release in nano-prodrug systems, emphasizing the importance of stimulus-response mechanisms such as pH, temperature, and tumor-specific enzyme activity. The challenge lies in aligning the timing and location of drug release with the arrival of nanoparticles at the tumor site to maximize therapeutic efficacy and minimize off-target effects. **(B)** This section highlights the complexity of integrating multiple stimuli, such as pH sensitivity with light or ultrasound responsiveness, to enhance specificity. The coordinated interaction of these stimuli requires advanced research into complementary activation pathways and feedback-regulated systems for controlled drug release. **(C)** The figure addresses the inherent variability in tumors and the TME, which complicates the design of universal nano-prodrug systems. Personalized approaches, including tumor-specific nanoparticle modifications and ligand-targeted delivery, are essential for improving precision and efficacy. **(D)** The final section outlines the hurdles in transitioning nano-prodrugs from preclinical research to clinical practice, including reproducibility, large-scale manufacturing, regulatory approval, and the need for standardized protocols and multicenter clinical trials to ensure safety and efficacy.

The application of nano-prodrugs presents significant potential in improving drug delivery and therapeutic outcomes, particularly in oncology. However, several challenges remain that hinder their widespread clinical application and efficacy (Fig. 7). Firstly, the precise control of drug release in nano-prodrug systems requires both accurate stimulus-response mechanisms and optimized spatiotemporal regulation. Achieving selectivity in drug activation is essential, ensuring that the nano-prodrug responds only to specific stimuli, such as pH, temperature, or enzyme activity, that are elevated in the tumor microenvironment [118]. However, beyond this specificity, a key challenge is synchronizing drug activation with tumor site localization and treatment timing. While a system may be designed to respond to a particular stimulus, the timing and location of drug release must be precisely coordinated with the arrival of nanoparticles at the tumor site. Mismatches between the activation window and nanoparticle delivery can significantly reduce therapeutic efficacy. Therefore, an integrated approach is necessary, where both the selectivity of stimulus-response mechanisms and the coordination of release timing and location are tightly controlled to maximize therapeutic impact and minimize off-target effects. The design of nano-prodrugs could focus on more selective stimuli triggers that exhibit sharp gradients in tumor tissues, such as specific enzyme activity unique to cancer cells or exploiting the tumor's extracellular matrix. Additionally, the use of multi-layered coatings on nanoparticles could provide added layers of specificity, where the drug is only released when both the carrier surface and the TME meet precise conditions, ensuring that the prodrug remains inactive in healthy tissues [119,120].

Secondly, synergy between multiple stimulus-responsive mechanisms poses another challenge. Multi-stimuli-responsive nano-prodrugs aim to increase specificity by integrating multiple triggers, such as combining pH sensitivity with light or temperature responsiveness. However, the coordinated interaction of these stimuli can be difficult to optimize. For example, pH-responsive mechanisms might respond too quickly or too slowly, while light or ultrasound activation may not reach the target site in a controlled manner [121]. Achieving optimal synergy between multiple stimuli requires a deeper understanding of their combined effects on drug release kinetics and biological responses, which is still an area in need of refinement. Research into the cooperative mechanisms of multi-stimuli-responsive prodrugs needs to be advanced by developing nanoparticles that incorporate complementary activation pathways. One promising direction involves the combination of intrinsic and extrinsic triggers, such as utilizing both tumor-specific enzymes and external stimuli like light or ultrasound. Achieving precise coordination of these mechanisms may be facilitated by incorporating feedback-regulated systems that allow nanoparticles to adjust their behavior in response to multiple environmental cues, ensuring controlled drug release.

Thirdly, the inherent heterogeneity of tumors, both at the genetic and microenvironmental levels, presents a significant challenge for the effective application of nano-prodrugs in cancer therapy. Tumors are not only genetically diverse between individuals but also exhibit intratumoral variability, with distinct regions demonstrating different molecular, physical, and metabolic characteristics. This variability complicates the design of universal nano-prodrug systems that can consistently target tumor cells across different patients and tumor sites [122]. Additionally, the TME, which varies between individuals and even within different areas of the same tumor, plays a crucial role in the response of nano-prodrugs to external stimuli such as pH, temperature, or enzyme activity [123]. Such diversity in tumor biology and the TME can lead to inconsistent drug release, limiting the efficacy of nano-prodrugs. To address this challenge, a more personalized approach is needed for the design and application of nano-prodrugs. Personalized medicine aims to tailor therapeutic strategies based on an individual's unique tumor characteristics, including genetic mutations, protein expression profiles, and TME conditions. For nano-prodrugs, this means developing systems that can respond to the specific biomarkers and environmental cues of a given tumor. Strategies such as tumor-specific nanoparticle surface modifications, ligand-targeted delivery, and the use of smart nanoparticles that can sense and adapt to varying TME conditions are critical in enhancing the precision and efficacy of nano-prodrugs.

Finally, the clinical translation of these systems is still in its nascent stage, with very few reaching clinical trials. The complexity of developing nanoparticles that are both safe and effective in humans presents significant hurdles. Issues such as reproducibility, large-scale manufacturing, regulatory approval, and safety concerns regarding long-term exposure to nanoparticles remain unresolved. Additionally, while preclinical models have shown promising results, clinical trials for nano-prodrugs are limited, and the transition from laboratory to clinic is fraught with challenges [4]. The difficulty in establishing reliable clinical outcomes, coupled with the lack of standardized protocols for nanoparticle use, further complicates the widespread adoption of this technology in medical practice to address the challenges of clinical translation, efforts should be focused on standardizing nanoparticle production and developing reliable and reproducible manufacturing processes that meet regulatory standards. Conducting multicenter clinical trials that evaluate the safety and efficacy of nano-prodrugs in diverse patient populations will be crucial for generating evidence to support their clinical adoption. Moreover, collaboration between academic institutions, industry, and regulatory bodies will be essential to accelerate the pathway from laboratory research to clinical application.

## 7 Conclusion

**Fig. 8:**
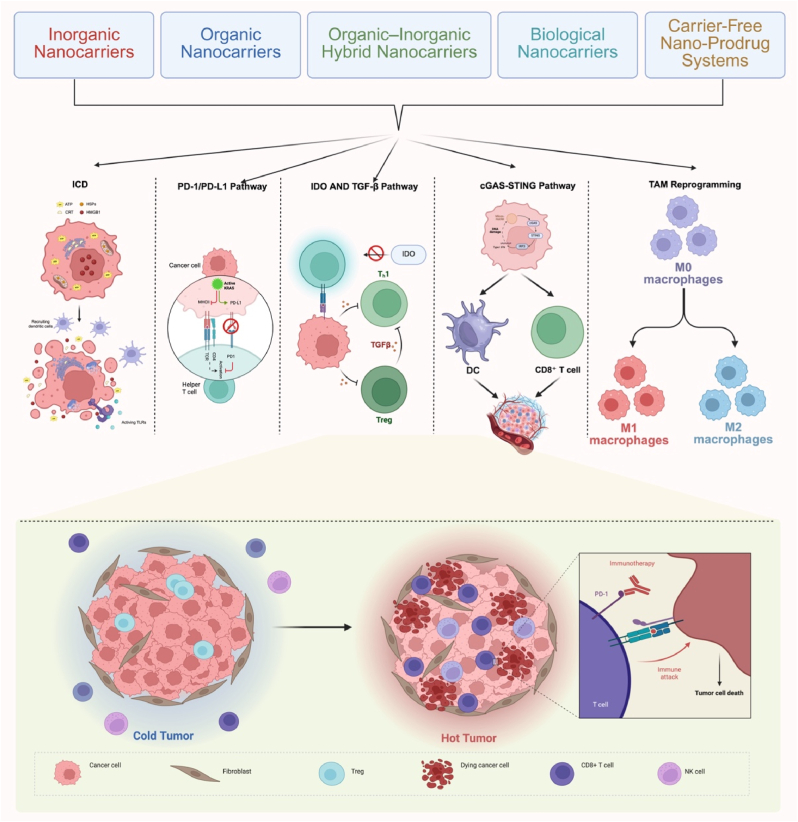
Mechanisms of nanoprodrug-mediated immune activation in cancer.

The integration of nanoprodrugs in immunotherapy offers a transformative approach to cancer treatment, with the potential to address many current limitations in therapeutic efficacy. Despite notable advancements, further refinement of nanoparticle-based delivery systems is essential to overcome challenges related to drug stability, targeted delivery, and immune modulation. Future innovations in nanomaterial design, as well as the development of more sophisticated strategies for overcoming tumor resistance mechanisms, will be crucial in translating nanoprodrug therapies into clinical success. With continued research, nanoprodrugs could significantly expand the therapeutic options available for patients, enabling more effective and personalized cancer treatments**(**Fig. 8).

Nanoprodrugs, by inducing ICD, releaseTAAs and DAMP), which activate DCs and stimulate T cell-mediated immune responses. Additionally, nanoprodrugs can modulate immune checkpoints such as the PD-1/PD-L1 pathway, overcoming immune suppression and restoring T cell function to promote anti-tumor immunity. The activation of the cGAS-STING pathway by nanoprodrugs enhances the production of type I interferons, which further stimulate DC activation and the infiltration of CD8^+^ T cells into tumors. Nanoprodrugs also target immune-suppressive factors such as IDO and TGF-β, boosting T cell activity. Furthermore, nanoprodrugs can reprogram TAMs from an immunosuppressive M2 phenotype to a pro-inflammatory M1 phenotype, increasing the immune response. Together, these effects facilitate the transition from cold tumors, which are characterized by limited immune infiltration, to hot tumors, where effective immune activation and anti-tumor immunity occur.

## CRediT authorship contribution statement

**Yunfan Lin:** Writing – original draft, Visualization. **Pei Lin:** Writing – original draft, Visualization. **Xu Chen:** Writing – review & editing, Writing – original draft. **Xinyuan Zhao:** Writing – review & editing, Writing – original draft, Funding acquisition. **Li Cui:** Writing – review & editing, Writing – original draft, Supervision, Funding acquisition.

## Ethics approval and consent to participate

Not applicable.

## Consent for publication

All authors gave their consent for publication.

## Funding

This work was supported by 10.13039/501100001809National Natural Science Foundation of China (82372905, 81901006), Young Top-notch Talent of Pearl River Talent Plan (0920220228), Guangdong Provincial Science and Technology Project Foundation (2022A0505050038), Science and Technology Program of Guangzhou (No.2025A04J3464), and Science Research Cultivation Program of Stomatological Hospital, 10.13039/501100010096Southern Medical University (PY2020002, PY2022019).

## Declaration of competing interest

The authors declare that they have no known competing financial interests or personal relationships that could have appeared to influence the work reported in this paper.

## Data Availability

No data was used for the research described in the article.
